# Optical Imaging of Retinotopic Maps in a Small Songbird, the Zebra Finch

**DOI:** 10.1371/journal.pone.0011912

**Published:** 2010-08-04

**Authors:** Nina Keary, Joe Voss, Konrad Lehmann, Hans-Joachim Bischof, Siegrid Löwel

**Affiliations:** 1 Lehrstuhl Verhaltensforschung, Universität Bielefeld, Bielefeld, Germany; 2 Institut für Allgemeine Zoologie und Tierphysiologie, Friedrich-Schiller-Universität Jena, Jena, Germany; Lund University, Sweden

## Abstract

**Background:**

The primary visual cortex of mammals is characterised by a retinotopic representation of the visual field. It has therefore been speculated that the visual wulst, the avian homologue of the visual cortex, also contains such a retinotopic map. We examined this for the first time by optical imaging of intrinsic signals in zebra finches, a small songbird with laterally placed eyes. In addition to the visual wulst, we visualised the retinotopic map of the optic tectum which is homologue to the superior colliculus in mammals.

**Methodology/Principal Findings:**

For the optic tectum, our results confirmed previous accounts of topography based on anatomical studies and conventional electrophysiology. Within the visual wulst, the retinotopy revealed by our experiments has not been illustrated convincingly before. The frontal part of the visual field (0°±30° azimuth) was not represented in the retinotopic map. The visual field from 30°–60° azimuth showed stronger magnification compared with more lateral regions. Only stimuli within elevations between about 20° and 40° above the horizon elicited neuronal activation. Activation from other elevations was masked by activation of the preferred region. Most interestingly, we observed more than one retinotopic representation of visual space within the visual wulst, which indicates that the avian wulst, like the visual cortex in mammals, may show some compartmentation parallel to the surface in addition to its layered structure.

**Conclusion/Significance:**

Our results show the applicability of the optical imaging method also for small songbirds. We obtained a more detailed picture of retinotopic maps in birds, especially on the functional neuronal organisation of the visual wulst. Our findings support the notion of homology of visual wulst and visual cortex by showing that there is a functional correspondence between the two areas but also raise questions based on considerable differences between avian and mammalian retinotopic representations.

## Introduction

In mammals the image of the visual environment, projected onto the retina by the eye lens, is represented in a topographical manner in a variety of noncortical (e.g. [1]) and cortical areas (e.g. [2]–[4]). Area 17, the first cortical station of the mammalian geniculocortical visual pathway, is one of the most intensely investigated areas in the brain. It has been shown that the topographic representation within area 17 is distorted due to a variety of reasons. First, as known from cartography, projecting an image to another curved surface (from the almost spherical retina to the complicated folds of the cortical surface) leads to distortions whereby the overall integrity of the map is preserved. Second, the fovea is more strongly represented than the periphery, due to a much higher density of ganglion cells sending axons into the brain [5]. Third, each hemisphere contains superimposed representations of both eyes (e.g. [6], [7]). Finally, computational maps [8] of orientation selectivity, directional sensitivity, or colour coding can further complicate the cortical mappings (e.g. [9]–[15]).

The mammalian cortex is mainly a thin layered structure on the surface of the forebrain. Areas with different sensory input and tasks form a two dimensional patchwork array first described in 1909 by Korbinian Brodmann [16]. In contrast, its homologue in birds is organised in three-dimensional clusters of neurons (nuclei) located at different positions within the forebrain [17]. These clusters are, however, organised similarly to cortical areas in that there is an input layer from which information is then spread distally and proximally [18]. However, a topographic organisation of these areas has been shown only in a few cases. For the telencephalon, it is the so called visual wulst, which - in the owl - has been shown to be topographically organised.

The visual wulst (hyperpallium) is part of one of the two main visual projections in birds, the so called thalamofugal visual pathway. It receives visual input from the retina via the nucleus geniculatus lateralis pars dorsalis of the thalamus. In zebra finches (*Taeniopygia guttata castanotis,* GOULD) it comprises several layered subunits named Hyperpallium apicale (HA), Nucleus interstitialis hyperpallii apicale (IHA, a narrow strip of neurons located along the lamina frontalis suprema (LFM)) and Hyperpallium densocellulare (HD) from dorsal to ventral. In the barn owl (*Tyto alba*) wulst neurons are suggested to be retinotopically organised [19]–[23], and exhibit an ordered arrangement of stimulus orientation preference [21], [22]. These features, as well as selectivity for direction of movement and binocular interaction [19], [21] are very similar to those found in the visual cortex of mammals [22]. Owls, however, are an exception among birds in that they have frontally placed eyes and a large binocular overlap. In the vast majority of birds, the eyes are placed laterally, and the binocular overlap of their visual field is fairly small (zebra finch: [24]; pigeon: [25]). The visual wulst receives input almost exclusively from the contralateral eye (zebra finch: [26], [27]; pigeon: [28]) although there is anatomical evidence for input also from the ipsilateral eye (e.g. [29]). Topographic representation of visual space was suggested for the visual wulst of domestic chickens (*Gallus gallus domesticus*) [30] on the basis of single neuron recordings.

The optic tectum of birds is homologous to the superior colliculus of mammals (e.g. [31]). It is the primary relay station of the tectofugal pathway and receives information only from the contralateral eye, due to the complete crossing of the optic nerves to the contralateral hemispheres. From the optic tectum, visual information is transferred to the thalamic nucleus rotundus, which, in turn, projects upon the entopallium of the telencephalon. From there, information is thought to reach the nidopallium and finally the arcopallium, which projects back to the optic tectum, thus completing a “tectal loop” [18]. It has also been shown that the visual wulst affects processing of visual information within the tectofugal pathway by projection to the optic tectum and the entopallium [32], [33]. The retinotectal projection has become a prime example of a retinotopic organisation and is widely used as a model for neuronal pathfinding in developmental biology (e.g. [34]). Within the tectum of the owl, the retinotopic map is in register with a map of acoustic space, representing the directions from which the owl perceives sounds [35]. Besides the developmental studies which were mainly performed on the chick, not much information is available on the topographic arrangement of neurons within the tectum of laterally eyed birds. Electrophysiological recordings have indicated a topographic representation of visual space in both, the pigeon [36]–[40] and the zebra finch [41], but high resolution information is lacking.

While single cell recordings allow a detailed characterisation of neuronal response properties like receptive field sizes and locations or orientation, direction, and colour preference, they are less ideal for analysing the fine-grained topographic organisation of a given brain area. Optical imaging of intrinsic signals [42] offers several advantages over conventional electrophysiological and anatomical techniques: A relatively large region of the brain can be visualised at the same time and activity patterns induced by different stimuli in the same cortical area can be obtained. Improvements introduced by Kalatsky and Stryker [43] reduced acquisition time and provided more natural stimulus conditions. The disadvantage of this method is its restriction to superficial brain areas.

In the current experiment, we used intrinsic signal optical imaging for the first time in a small, laterally eyed bird, the zebra finch (*Taeniopygia guttata castanotis,* GOULD), to visualise topographic organisation of both the visual wulst of the thalamofugal visual system and the optic tectum of the tectofugal pathway. The retinotopic maps we obtained in our experiments revealed previously unknown details of the organisation of the two brain areas. The results on the optic tectum confirmed conclusions drawn earlier on the basis of anatomical tracing studies and electrophysiological experiments. For the visual wulst, our experiments revealed, among other details, the existence of more than one retinotopic representation, a finding which has implications on the homology of avian and mammalian visual areas.

## Materials and Methods

20 zebra finches of both sexes from the breeding stock of the Bielefeld Behavioural Biology Department were used for this study. All experimental procedures were performed according to the German Law on the Protection of Animals and had been approved by the local government, *Landesamt für Natur, Umwelt und Verbraucherschutz Nordrhein-Westfalen*, approval number AZ 9.93.2.10.36.07.105.

Animals were anaesthetised with 0.12 ml 20% urethane injected intramuscularly and kept under red light to maintain body temperature. The feathers surrounding the ear holes were plucked and local anaesthesia (2% xylocaine jelly) was applied. The birds were then fixed in a stereotaxic headholder for small birds [44]. For exposure of the visual wulst, the head feathers were removed and xylocaine jelly was applied on the scalp. The skull of one hemisphere was exposed and a craniotomy was performed leaving the dura mater intact. The cranial window exposing the visual wulst extended from approximately 7.1 mm–1.2 mm anterior of the reference point (y-point; meeting point of cerebellum and both hemispheres) and extended as far as 4.9 mm laterally.

For recordings from the optic tectum, a lateral approach was used. Due to its location only the most lateral region of the optic tectum could be exposed without compromising the stability of the skull and its fixation to the head holder. The dimensions of the uncovered tectal region were approximately 2.2 mm×1.1 mm.

Low melting point agarose (2.5% in saline) and a glass coverslip were placed over the exposed area to protect the surface of the brain from desiccation and to prevent refraction errors caused by an uneven surface. After coagulation of the agarose, a scalpel was used to remove the overlapping parts at the edges of the glass coverslip. Using customary correction fluid (LACO Office Products, Germany) the borders of the agarose block were then coated to prevent it from drying and to avoid illumination of the brain from the side.

The contralateral eye was opened by retraction of the lower eye lid. To ascertain whether the nictitating membrane was still intact, the inner corner of the eye was tactically stimulated. If the nictitating membrane did not close over the eyeball, silicone oil was used to prevent drying of the eye.

**Figure 1:**
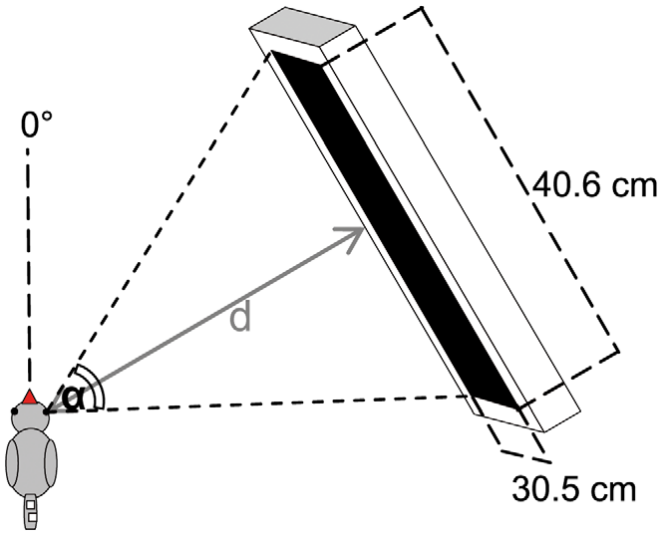
Location of the visual stimulus. The stimulus monitor was positioned at 60° from midline. Depending on the distance **d** between the animal and the monitor visual stimuli cover different proportions (angle **α**) of the visual field. All retinotopic maps shown in the following figures (3 to 8) were recorded using the indicated experimental configuration. Values for monitor distance d and angle α are indicated in the figure legends for each figure separately.

Neuronal activity in the zebra finch visual system was recorded using the intrinsic signal optical imaging method initially developed by Grinvald et al. [42] and recently modified by Kalatsky and Stryker [43]. Using a temporally periodic stimulus and Fourier analysis to extract the response at the stimulus frequency, retinotopic maps were visualised in less than 10 min. In short, a high refresh rate monitor (Hitachi Accuvue HM 4921-D, 85 Hz) was placed at an angle of 60° from midline in front of the bird's contralateral eye. In some experiments, monitor location was varied between 0° and 90°, or the monitor was placed at 60° on the ipsilateral side. The distance between eye and monitor varied between 25 cm and 40 cm, so that visual angle varied between 80° and 54° azimuth and 60° and 40° elevation (Figure 1).

Previous electrophysiological studies examining the characteristics of wulst neurons in pigeons (e.g. [45]) have found moving stimuli to be most effective in driving neurons. Although moving light spots of varying diameters were also effective in eliciting neuronal activation, optimal responses were generated with straight edges or bars oriented perpendicular to their axis of movement. A substantial proportion (about 70%) of tectal neurons is sensitive to moving stimuli and about 30% of these show a high degree of directional specificity [46]. However, most tectal units are only broadly tuned for direction and the majority prefer forward or downward directions of movement [47], [48]. Generally, the stimulus used in our study was thus appropriate to elicit reliable responses in any direction. It was either a horizontal white bar on a black background drifting up or down to reveal elevation maps, or a vertical bar drifting left or right to reveal azimuth maps. The bars, generated by a Matrox G450 board (Matrox Graphics®, Inc., Quebec, Canada) controlled by custom software, were 2°–4° wide and moved with a speed of 7 to 10°/sec. Since bars were moved in two opposing directions, a total of 4 stimulus conditions (vertical bars moving left and right, horizontal bars moving up and down) was employed. The neuronal responses of the visual wulst and the optic tectum to these visual stimuli were recorded using a Dalsa 1M30 CCD camera (Dalsa, Waterloo, Canada), controlled by custom software, placed above the bird's head. A 135 mm×50 mm tandem lens configuration (Nikon, Inc., Melville, NY) was used to monitor a brain area of 4.6 mm×4.6 mm. An image of the surface vascular pattern was taken under illumination of the brain with green light of 550±3 nm wavelength. Neuronal activity was recorded by illuminating the brain with red light of 610±3 nm wavelength. The camera was focussed at a depth of 500 µm below the pial surface and frames were acquired at a rate of 30 Hz. Four subsequent frames were temporally averaged, resulting in the storage of 7.5 frames/sec. The original spatial resolution of 1024×1024 pixels was also reduced to 512×512 pixels (2×2 binning) before data storage.

Elevation and azimuth maps of the contralateral visual field were calculated from the acquired frames by Fourier analysis to extract the signal at the stimulation frequency using custom software as described previously [43], [49]. While the phase component of the signal was used for the calculation of retinotopy, which is depicted in so-called phase maps, the amplitude component represents the intensity of neuronal activation, i.e. response magnitude (expressed as fractional change in reflectance×10^4^) in magnitude maps. The magnitude of the most intensely activated pixel within the observed neuronal activity patch is included as a number in each magnitude map shown. Retinotopic maps were colour coded so that neuronal activation within the brain area observed could be correlated with the position of the stimulus on the monitor inducing that activation. The combined information of the magnitude of neuronal activation and retinotopy is displayed in so-called polar maps.

In order to assess the preservation of visual field proportions in the retinotopically activated brain areas, iso-azimuth and iso-elevation lines were linked to specific stimulus positions and then superimposed on the images taken. The spacing between iso-azimuth or iso-elevation lines was always 10° of visual angle. An increasing distance between animal and stimulus monitor resulted in a decrease of visual space occupied by the monitor and therefore a decline in the number of isolines passing over it. A Matlab routine was used to calculate the dimensions of a given map by selecting all pixels that showed at least 30% of the responsiveness of the most responsive pixel in the map. Then the mean size of visual wulst and optic tectum activity patches and their standard deviations were calculated. The excised activity patch was used to crop the images with superimposed isolines to obtain images with patch confined isolines. The spacing between isolines on the brain surface was measured in order to reveal possible differences in magnification factor. From each bird one azimuth and one elevation map were used for a measurement near the foveal representation and two measurements in the periphery. As it was not possible to evaluate the parameters of all of the maps, the sample sizes for these calculations differ from n = 14. In order to visualise the location of the neuronal activations within the visual wulst, electrical lesions (90V, 30 µA) were made at the coordinates of the retinotopic representation in three zebra finch brains. Following transverse sectioning of each brain, slices were mounted and Giemsa-stained. Data are given as means ± standard deviation.

## Results

Our experiments confirm and extend previous results concerning the retinotopic organisation of the optic tectum and in addition show that the visual wulst (hyperpallium) also contains a retinotopic representation of the visual world. The most apparent difference between both representations is the spatial extension of the maps and the more complicated structure of the visual wulst map (see below). Not all imaging experiments resulted in identical topographic maps as one would have expected if the topography were based on hard-wired connectivity from the retina to the target region. Elevation maps, especially, showed some variation. Likewise, there were cases in which only the azimuth and not the elevation maps were clear enough to be used for quantitative evaluation, and vice versa. This causes the different n's for the calculations below.

### Optic Tectum

**Figure 2:**
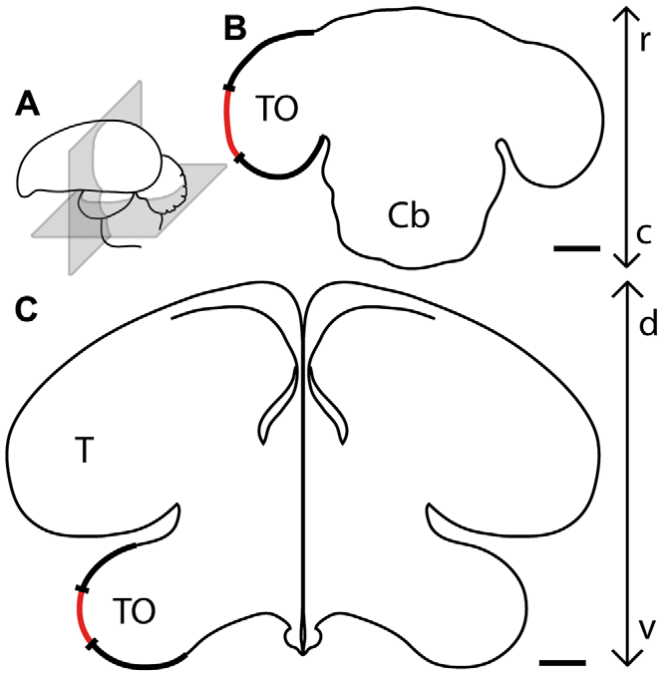
Position and extent of the optically imaged tectal surface. (A) Schematic drawing of the zebra finch brain indicating the location of horizontal (B) and transverse (C) sectional planes. In (B) and (C), the left optic tectum is highlighted (thick black line) and the position and extent of the optically imaged tectal surface is indicated by the red line. Cb  =  Cerebellum, T  =  Telencephalon, TO  =  Tectum opticum. c, caudal; d, dorsal; r, rostral; v, ventral. Scale bar  = 1 mm.

Tectal activity was imaged in 4 zebra finches. The exposed area was located quite centrally on the tectal surface (Figure 2) and extended about 1.1 mm in the dorsoventral and 2.2 mm in the rostrocaudal axis (n = 4, 2.42±0.81 mm^2^, about 6% of the entire tectal surface.)

**Figure 3:**
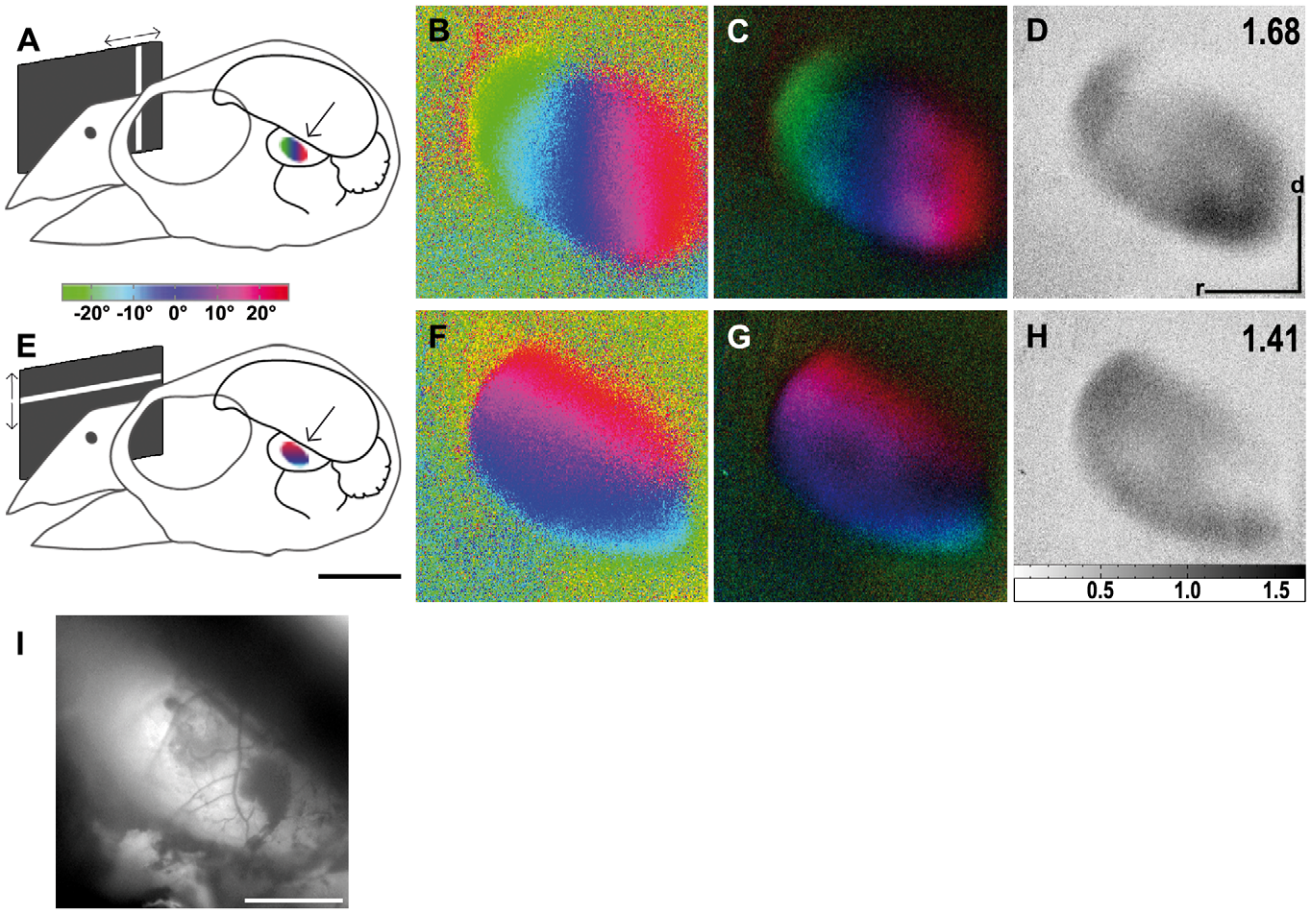
Retinotopic maps recorded with intrinsic signal optical imaging in the optic tectum. (A and E) Schematic illustration of the visual stimulus and a colour coding used to visualise the tectal response (arrows) with either vertical (azimuth stimulus, A) or horizontal moving bars (elevation stimulus, E). Colour-coded phase (B, F) and polar maps of retinotopy (C, G) and grey-scale-coded response magnitude maps (D, H) are illustrated. The magnitude of the optical response is illustrated as fractional change in reflection×10^4^: the darker the activity patch the more active was the corresponding brain region. The absolute magnitude of the darkest spot within the activity patch is given as a number in the upper right corner in the graphs. Neuronal activity elicited by visual stimulation with moving vertical (B–D) and horizontal bars (F–H) are illustrated. Note the identical orientation of the tectal map and the presented visual stimulus with a slight tilt of the elevation map against the horizontal axis. Since the borders of the activity patch were also the borders of the exposed tectal surface, the edges of the patch were very well defined in the displayed images. In (I) the surface vascular pattern corresponding to the representation in (B) to (D) and (F) to (H) is shown. d, dorsal; r, rostral. Scale bar in (A) and (E)  = 5 mm. Scale bar in (B) to (D), (F) to (H) and (I)  = 1 mm. Monitor distance d  = 40 cm, α = 54° (see Figure 1).

Figure 3 illustrates tectal activity maps recorded after visual stimulation of the zebra finch with moving vertical (Figure 3A) or horizontal bars (Figure 3E). The retinotopic activation in azimuth maps (Figures 3B to D) extended from the rostral to the caudal margin of the exposed brain area whereas neuronal activity in elevation maps (Figures 3F to H) stretched from the dorsal to the ventral margin. Although the azimuth and the elevation stimulus lines were exactly perpendicular to each other, the angle between the colour codings of the two maps was obviously different from 90°. While vertical seemed to be represented also in a vertical direction on the tectum, the tectal representation of the visual horizon was tilted to a certain degree (Figure 3F).

**Figure 4:**
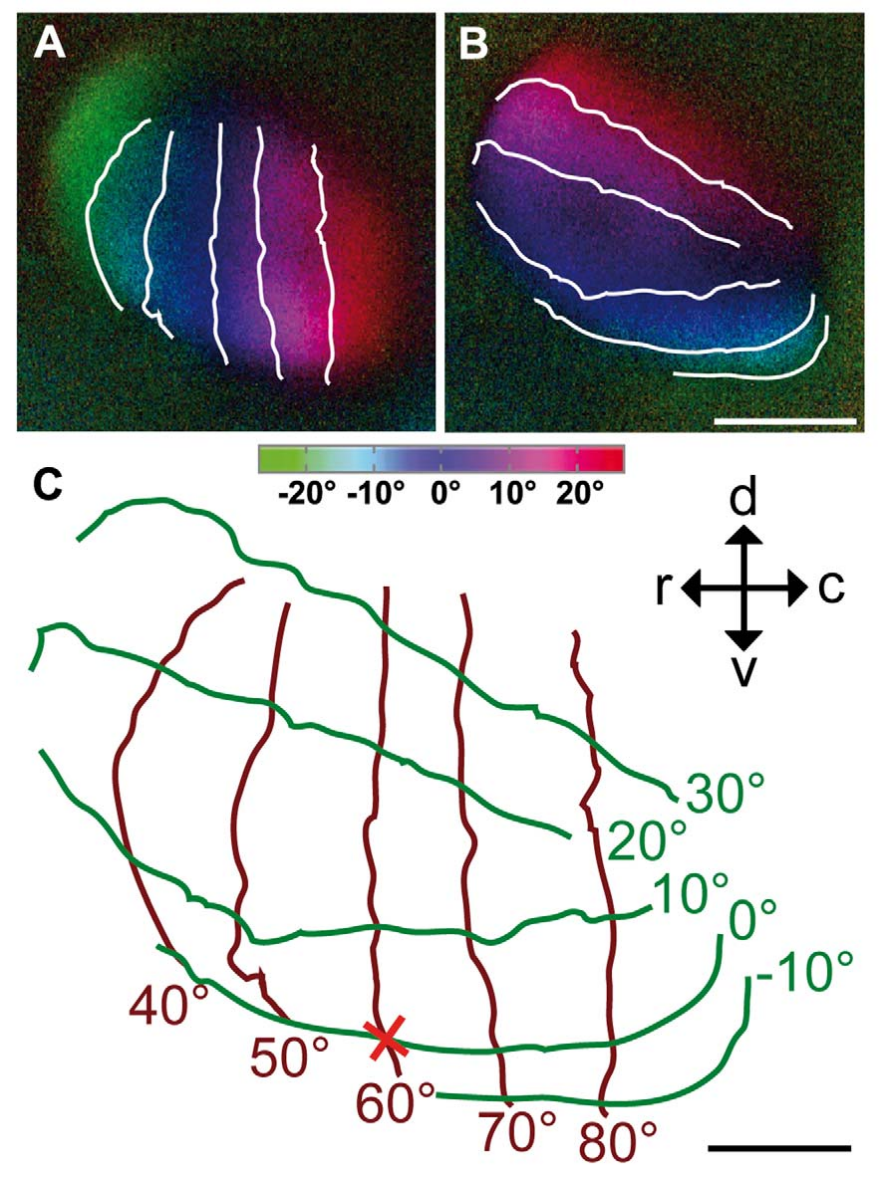
Colour-coded polar maps of retinotopy in the optic tectum superimposed with iso-azimuth and iso-elevation lines. An azimuth (A) and an elevation retinotopic polar map (B) are superimposed by iso-azimuth and iso-elevation lines indicating the alignment of visual field vertical and horizontal contours on the surface of the brain (C). The position of the fovea is indicated by a red cross. Iso-azimuth is plotted by brown lines, iso-elevation by green lines. The contours overlying the images have a spacing of 10°. The isolines drawn over dark blue regions correspond to lines running through the centre of the stimulus monitor (0° on colour coding). The monitor was horizontally centred at the angle of the fovea (60°), but the horizontal through the bird's eye lay 9° below the horizontal centre line of the monitor, consequently, so does the fovea in (C). The inset in (C) indicates the orientation of the activity patches within the optic tectum, viewed from the side. c, caudal; d, dorsal; r, rostral; v, ventral. Scale bar (A) and (B)  = 1 mm. Scale bar (C)  = 500 µm. Monitor distance d  = 40 cm, α  = 54° (see Figure 1).

In Figure 4, we superimposed iso-azimuth and iso-elevation lines on the retinotopic maps of the tectum to illustrate the degree of preservation of visual field proportions. It was determined earlier [24], [50] that the foveal axis of the anaesthetised zebra finch is directed to an angle of 62° from the beak horizontally and that its elevation is about 0° (“looking” at the horizon). This allowed marking the position of the foveal representation (red cross). Spacing between isolines near the foveal representation and isolines in the periphery of the activity patches did not significantly differ from each other, probably because of the small part of the tectal representation of the visual field which we were able to expose, comprising about ±20° horizontally and +30°/−10° vertically. The measurements of both areas were thus taken together. According to these measurements, a difference of 10° azimuth was mapped onto 355 µm±118 µm brain surface (n = 4), and 10° difference in elevation were mapped onto 360 µm±100 µm tectal surface (n = 3). As was already indicated by the colour coded maps (Figure 3F), the iso-elevation lines were not exactly perpendicular (90°) to the azimuth lines, but were tilted against the horizontal by about 20°.

### Visual Wulst

In the visual wulst, patches of increased neuronal activity after visual stimulation were recorded in 14 of 16 examined birds. In these experiments it was possible to expose and visualise almost the entire forebrain surface, extending from about 1 to 7 mm anterior and from the midline to 5 mm lateral, as measured from the y-point [51]. The activity patches induced by the moving visual stimuli were much smaller than those recorded in the optic tectum. On average, the activated area was 0.46±0.17 mm^2^ (n = 14) extending about 0.75±0.19 mm mediolaterally and 0.81±0.20 mm rostrocaudally (n = 14). The centre of the map was 3670 µm±460 µm anterior and about 2370 µm±350 µm lateral (n = 10) to the y-point. In general, the stimulation with vertical stripes moving horizontally (azimuth maps) resulted in most cases in images showing a clear topography. By contrast, images resulting from stimulation with horizontal stripes moving vertically (elevation maps) were much more variable and did not span all elevations examined (see below).

**Figure 5:**
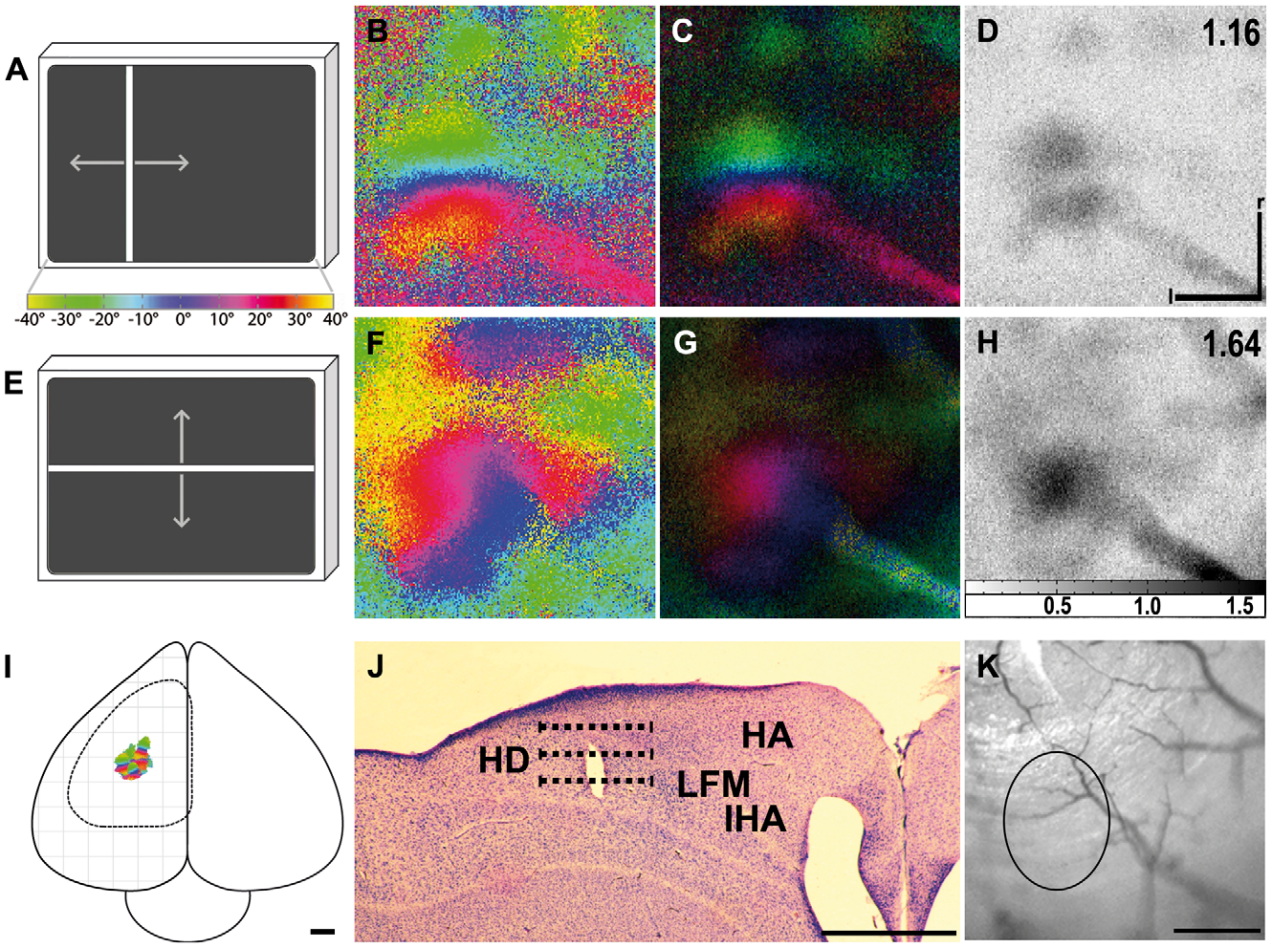
Retinotopic maps recorded with intrinsic signal optical imaging in the visual wulst. (A and E) Schematic illustration of the visual stimulus and a colour coding used to visualise the wulst response with either vertical (azimuth stimulus, A) or horizontal moving bars (elevation stimulus, E). Colour-coded phase (B, F) and polar maps of retinotopy (C, G) and grey-scale-coded response magnitude maps (D, H) are illustrated. The magnitude of the optical response is illustrated as fractional change in reflection×10^4^: the darker the activity patch the more active was the corresponding brain region. The absolute magnitude of the darkest spot within the activity patch is given as a number in the upper right corner in the graphs. Neuronal activity elicited by visual stimulation with moving vertical (B–D) and horizontal bars (F–H) are illustrated. In the elevation phase map (F) the lack of green and yellow regions in the retinotopic representation indicates that only part of the stimulated visual field elicits neuronal activation in the wulst. In all images (B to D and F to H) a blood vessel stretching from the lower right corner toward the centre of the activation is distinguishable, producing an artefact clearly visible in the polar maps (C, G) and the magnitude maps (D, H). In (I), six exemplary optically imaged retinotopic maps in the visual wulst are illustrated on a schematic whole zebra finch brain, viewed from above. The dashed line indicates the contour of the exposed forebrain surface. A Giemsa-stained transverse section at 3900 µm anterior to the y-point with an electrical lesion (bright spot) set at the position of the retinotopic map ((B) to (D) and (F) to (H)) is indicated in (J). The representation was positioned about 2300 µm lateral from midline and was about 800 µm wide (dashed lines). Focus depths, in which we recorded identical mappings, were 300 µm, 500 µm and 700 µm, corresponding to the upper, the middle and the lower dashed line. The representation is positioned at the borders between Hyperpallium apicale (HA), Nucleus interstitialis hyperpallii apicale (IHA) and Hyperpallium densocellulare (HD), probably even more in HD. IHA is a narrow strip of neurons along the lamina frontalis suprema (LFM). In (K) the surface vascular pattern corresponding to the representation in (B) to (D) and (F) to (H) is shown. The circle indicates the location of the representation and the above mentioned blood vessel can be seen in the lower right corner of the image. l, lateral; r, rostral. Scale bar in (B) to (D), (F) to (H) and (K)  = 500 µm. Scale bar in (I) and (J)  = 1 mm. Monitor distance d = 25 cm, α = 80° (see Figure 1).

Figure 5 shows an example of an activation patch obtained in the visual wulst. Drifting vertical bars (Figure 5A), testing the activation at different azimuth levels, elicited neuronal activity extending rostrocaudally in the wulst: More frontally positioned visual stimuli activated the rostral part of the activated patch, whereas more lateral stimuli activated the caudal part (Figures 5B, C). Drifting horizontal bars, testing the activation at different elevation levels (Figure 5E), induced a retinotopic activation of wulst neurons which extended mediolaterally in the activated area: Lower parts of the visual field were represented more medially, upper parts more laterally in the zebra finch brain hemisphere (Figures 5F, G). As in the optic tectum, the azimuth maps were thus usually shifted in their orientation by approximately 90° compared to elevation maps. Figure 5 I illustrates the location and size of some of the maps (selected to show the variation) in relation to the whole zebra finch brain. An electrical lesion in a Giemsa-stained transverse section depicts the location of the activation patch within the wulst (Figure 5J). The three horizontal stripes depict the lateral extension of the topographical representations we obtained. The activation patch is located at the rim of the lamina frontalis suprema (LFM), and the topography could be detected at all depth as indicated by the horizontal lines (300 µm, 500 µm, 700 µm). Figure 5K depicts the surface vascular pattern of the recording area described in this figure. It indicates that the elongated activity pattern reaching from the lower right corner of the activation maps to the centre may be an artefact caused by a blood vessel.

**Figure 6:**
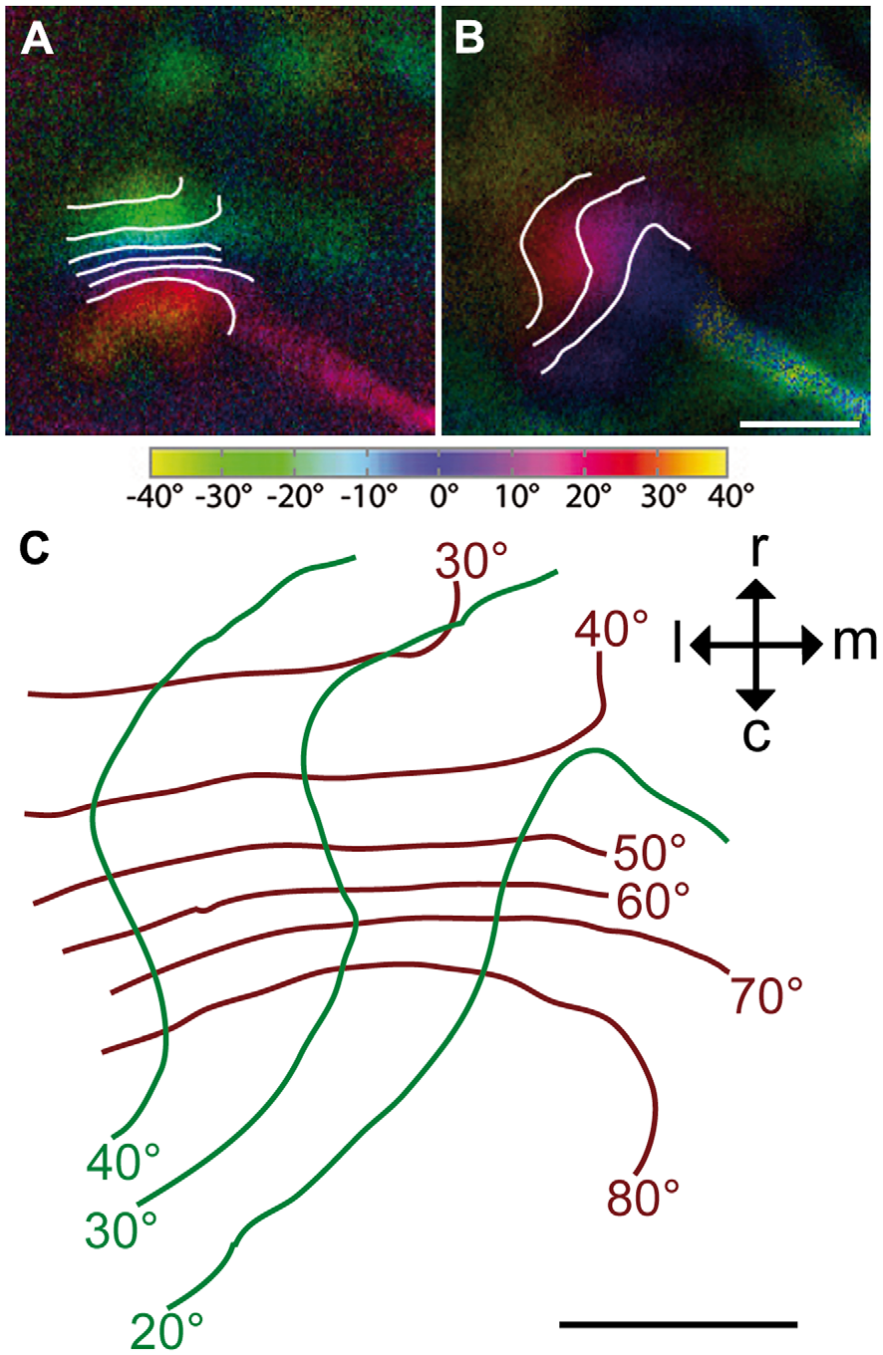
Colour-coded polar maps of retinotopy in the visual wulst superimposed with iso-azimuth and iso-elevation lines. An azimuth (A) and an elevation retinotopic polar map (B) are superimposed by iso-azimuth and iso-elevation lines (for further explanations see text). In (C) iso-azimuth is plotted by brown lines, iso-elevation by green lines. The contours overlying the images have a spacing of 10°. The isolines drawn over dark blue regions correspond to lines running through the centre of the stimulus monitor (0° on colour coding). As indicated by the red and blue colour coding in (B), the retinotopic elevation activity patch only represents the upper monitor half. The monitor was horizontally centred at the angle of the fovea (60°), but the fovea was directed at a position below the horizontal centre line of the monitor and can thus not be mapped. The inset in (C) indicates the orientation of the activity patches within the visual wulst, viewed from above. c, caudal; l, lateral; m, medial; r, rostral. Scale bar (A) and (B)  = 500 µm. Scale bar (C)  = 250 µm. Monitor distance d = 25 cm, α = 80° (see Figure 1).

**Figure 7:**
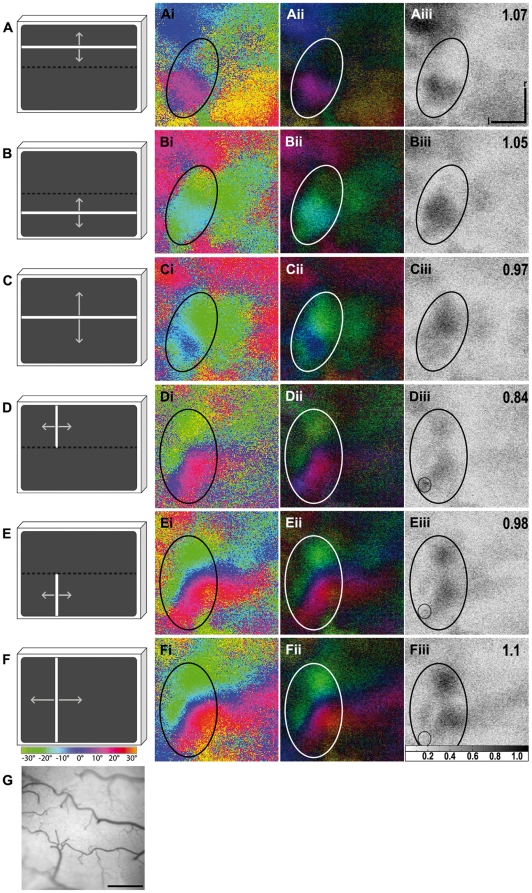
Activity elicited by stimulation at different elevations. In (A–C), three different elevation stimuli are illustrated, confined to either the upper (A) or lower part of the stimulus monitor (B) or extending over the entire monitor (C). In (D–F), the corresponding azimuth stimuli are illustrated: confined to the upper (D) or lower part of the monitor (E) or extending over the entire monitor (F). The colour coding used to visualise the wulst response is shown below the monitors. Colour-coded phase (Ai-Fi) and polar maps of retinotopy (Aii-Fii) and grey-scale-coded response magnitude maps (Aiii-Fiii) are illustrated. The magnitude of the optical responses is illustrated as fractional change in reflection×10^4^: the darker the activity patch the more active was the corresponding brain region. The absolute magnitude of the darkest spot within the activity patch is given as a number in the upper right corner in the graphs. Activity patches are marked by circles. An artefact is marked by a small circle in images (Diii) to (Fiii) and was excluded from the calculation of the absolute magnitude of these images. As can be seen from the magnitude maps with additional absolute magnitude values (Aiii) and (Biii) stimulation of the upper and also the lower monitor half with a vertically moving stimulus generated a strong neuronal response with corresponding colours displayed in the phase maps (Ai) and (Bi). Stimulation of the entire monitor, however, induced a weaker neuronal response (Ciii). The corresponding green and blue coloured activity patch in phase map (Ci) was lacking activation from the upper monitor half, represented by red colours. Stimulation of the upper monitor half with a horizontally moving stimulus induced a weaker neuronal response (Diii) than stimulation of the lower monitor half (Eiii), whereas stimulation of the entire monitor induced an even stronger neuronal response (Fiii). For further explanations see text. In (G) the surface vascular pattern corresponding to the representation in (Ai) to (Fiii) is shown. l, lateral; r, rostral. Scale bar in (Ai to Fiii) and (G)  = 500 µm. Monitor distance d = 30 cm, α = 68° (see Figure 1).

Although the mapping shown in Figures 5 and 6 was the most common one among our recordings, there were other examples (compare Figures 7C and F) where azimuth and/or elevation maps were different and not easy to interpret. Such variation was much more pronounced in elevation maps. As yet, we have not much information on how this variation is generated. For the calculation of the parameters below, we thus selected maps similar to that found in Figure 5. This causes the differences in the number of cases (12 of 14 of the azimuth, 5 of 14 of the elevation maps). In these maps, the representation of 10° visual space on the visual wulst in foveal and peripheral regions of azimuth and elevation maps was not different between fovea and periphery. 10° azimuth difference of visual space were mapped on 105 µm±50 µm brain surface (n = 12). Elevation differences of 10° visual space were mapped on 190 µm±80 µm brain surface (n = 5). Thus, vertically moving stimuli are represented on more brain tissue compared with horizontally moving stimuli. Figure 6 shows an example of the iso-azimuth and iso-elevation lines calculated from the azimuth and elevation map of one experiment demonstrating that in the horizontal (brown lines) the representation spans about 50° of visual space, while it is about 20° in the vertical (green lines).

All experiments described as yet were made with the monitor in a standard position (centre of the screen horizontally at 60° azimuth, vertically at 7°–10° elevation above the horizon, depending on the distance of the screen) in front of the contralateral eye. Most recordings were made from the left hemisphere. Optical images recorded from the standard position at the right hemisphere showed no difference to the left hemisphere recordings. Effects, however, on the magnitude of the responses were observed when the stimulus monitor was moved from the standard position to other locations. Ipsilateral stimulation (stimulus monitor placed at 60° in front of the left eye) yielded images without detectable neuronal activity. Visual stimuli presented in front of a bird at 0° in the binocular visual field also yielded no detectable neuronal activity upon stimulation of either eye (see also below).

When the monitor was moved from the frontal position (0°) to more lateral locations (30°, 60°, 90°), the activity patch on the wulst surface accordingly showed a rostrocaudal displacement. As in the experiment described above, frontal stimulation (0°) did not result in reliable activation patterns. Visual stimulation of all other positions reproducibly elicited topographic maps. The position of the activity patch shifted caudally if the stimulus was displaced from 30° to 60°, and further caudally after a stimulus displacement from 60° to 90°. The experiment also suggested that magnification of the map was strongest in the area around the fovea and became smaller with more frontal or more caudal positions of the stimulus monitor. However, this remains to be examined in more detail.

The effects of vertical displacement were somewhat more complicated (Figure 7) and offer additional information as to why we did obtain so much variation in the elevation maps. In the standard position (monitor distance to bird  = 30 cm; monitor centred at 60° laterally, vertically at 9° elevation above the horizon) we noticed that red and blue colours prevailed in elevation maps (see e.g. Figures 5F, G and 6B), representing stimulus positions in the upper and the middle part of the monitor. Yellow and green, representing visual stimuli in the top most and the lowest stimulus positions, were not observed. After lifting the monitor to a position in which the centre was situated 35° above the horizon, thus about 26° higher than in the standard position, the elevation maps displayed only blue and green colours while red was no longer visible (Figure 7C). This indicated that in the standard position the lower part of the screen did not contribute to the activation pattern of the visual wulst, while in the elevated position the upper part of the screen was not activating neurons within the wulst when horizontal stimuli were moved upwards and downwards. Taken together, this suggested that only stimuli within elevations from approximately 17° to 42° above the horizon elicited wulst activity. In contrast to the elevation maps, the azimuth maps did not show such a difference when the monitor was lifted: after visual stimulation with vertical bars, complete retinotopic maps were obtained in both monitor positions (compare Figures 5B and 7F).

To further assess this phenomenon, we designed an experiment in which visual stimuli were displayed only in the upper or lower half of the monitor. Although the experiment was performed only once, we report the results here because they indicate that the wulst maps are probably not simply one to one projections from the retina. During this experiment, the centre of the monitor was located 35° above the horizon. On the basis of the previous observations, we expected to find activation only when the stimulus passed over the lower half of the monitor. There was indeed a strong activation by horizontal lines passing vertically over the lower part of the monitor, as expected with blue and green colours representing that lower part of the stimulus monitor (Figures 7 Bi to Biii, activity index 1.05). However, there was also a strong activation when the stimulus was passing only the upper part of the monitor (Figures 7 Ai to Aiii, activity index 1.07). In this case, red and blue, the codes for the upper half of the monitor, were visible in contrast to the trial in which the stimulus passed over the entire monitor (Figures 7 Ci to Ciii). Also surprisingly, both activity patches were located at the same position on the wulst surface, and not displaced as would be expected.

In azimuth maps there was a stronger activation by the vertical stimulus running horizontally on the lower half of the monitor (Figures 7 Ei to Eiii, activity index 0.98). The activation was weaker when stimulation was restricted to the upper half of the monitor (Figures 7 Di to Diii, activity index 0.84). Again, the activation patches were located at the same position. This experiment suggests that the region of the visual field activating visual wulst most effectively reaches from about 17° to 42° above the horizon in elevation, and that there is some interaction between different parts of the topographic representation. However, this requires further detailed investigation in future experiments.

**Figure 8:**
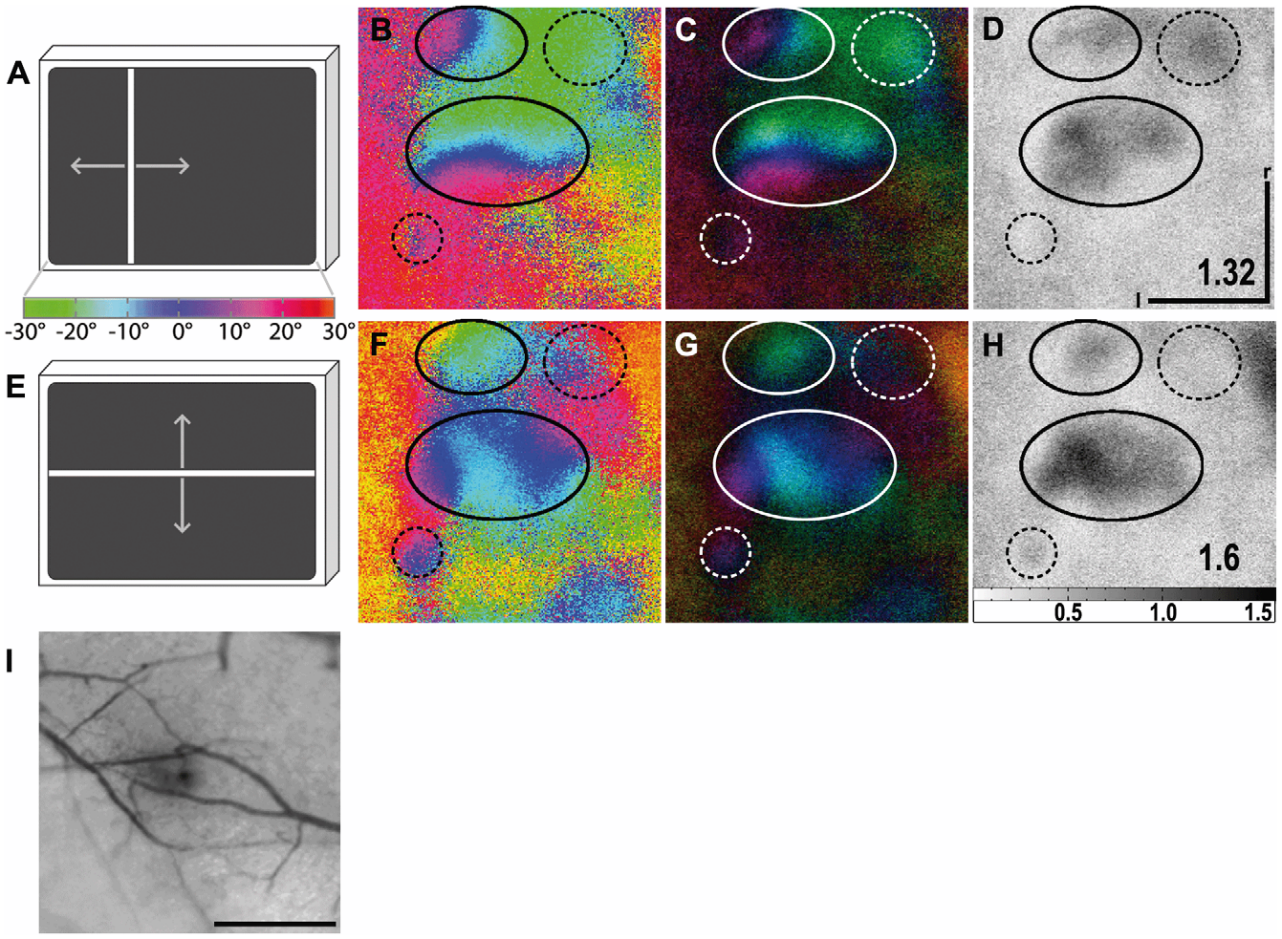
Maps of multiple visual areas recorded with intrinsic signal optical imaging in the visual wulst. (A and E) Schematic illustration of the visual stimulus and a colour coding used to visualise the wulst response with either vertical (azimuth stimulus, A) or horizontal moving bars (elevation stimulus, E). Colour-coded phase (B, F) and polar maps of retinotopy (C, G) and grey-scale-coded response magnitude maps (D, H) are illustrated. The magnitude of the optical responses is illustrated as fractional change in reflection×10^4^: the darker the activity patch the more active was the corresponding brain region. The absolute magnitude of the darkest spot within the activity patch is given as a number in the lower right corner in the graphs. Neuronal activity elicited by visual stimulation with moving vertical (B–D) and horizontal bars (F–H) are illustrated. Activity patches are marked by circles. Dashed circles mark the position of additional non topographical and weakly activated regions. In (B) two distinct retinotopic activity patches can be distinguished. A large, strongly activated one (see (D)) and a smaller, weaker one located anterior to the large patch and shifted in its relation to the large patch. In the elevation phase map (F) the strongly activated patch (see (H)) is also the larger of the two marked activity patches. The retinotopic colouring, however, somehow appears to be mirrored, which might indicate the large patch in (F), and therefore also that in (B), to be composed of two smaller patches. In (I) the surface vascular pattern corresponding to the representation in (B) to (D) and (F) to (H) is shown. l, lateral; r, rostral. Scale bar in (B) to (D), (F) to (H) and (I)  = 500 µm. Monitor distance d = 35 cm, α = 60° (see Figure 1).

The most unexpected and exciting finding of our wulst experiments was the appearance of multiple activity patches. In about two-thirds of all birds we found azimuth and elevation maps with more than one topographic representation. Figure 8 shows a typical example. A strongly activated patch was accompanied by a smaller and weaker activated one with a topographic map which was rotated against that of the strongly activated patch (Figures 8B, C). Some recordings even indicated up to five representations, but the activity patterns were not elicited reliably enough for additional quantification. The presence or absence of additional topographic representations did not seem to be correlated to overall response strength, since we observed activity patches in experiments with both high and lower maximum signal strength.

## Discussion

Our results clearly demonstrate that intrinsic signal optical imaging can be successfully used to visualise brain activity and retinotopic maps in small birds, such as the zebra finch. We were able to demonstrate a topographical representation of the visual world in both the optic tectum and the visual wulst. The resolution of the technique was good enough to even show the very small (∼750 µm diameter) topographical maps of the visual wulst in sufficient detail. While some of our results confirmed and extended previous findings obtained by anatomical or electrophysiological techniques, we also obtained completely new and unexpected details, especially for the visual wulst, the telencephalic target of the thalamofugal pathway.

Our setup was originally designed for optical imaging studies in mice in which it has been shown that moving bars are optimal for retinotopic mapping [43], [49]. Since the same stimuli were also shown to drive neurons within the avian wulst and the optic tectum (see above), we were able to use the same setup for our study. We indeed obtained quite reliable activity patterns in all cases. The habituation described in previous papers (e.g. [48]) for tectal neurons appeared to pose no problem for our stimulation schedule. We therefore did not address this issue in more detail.

As mentioned in the results section, the surface of the optic tectum could only be exposed for recording to a limited extent. As the direction of the foveal axis in anaesthetised birds [24], [50] is known, it was possible to centre the monitor to this foveal axis (60° azimuth, 0° elevation). Using the colour coding of the activity patterns, we were then able to locate the foveal representation on the optic tectum (see Figure 4). Within the trepanation, an area around the fovea of about ±20° azimuth and +30°/−10° elevation was visible. This is a quite small part of the whole visual field which in total spans about 170° in the horizontal plane [24] and approximately 120° in the vertical. Accordingly, our experiments do not exhibit the over-representation of the foveal region as it has been demonstrated electrophysiologically in the pigeon [52] and the zebra finch [53]. This discrepancy between the present and previous results could be due to the fact that the area examined was only a very small part of the entire tectal surface. According to a rough estimate, the size of the examined area was about 6% of the total tectal surface. As can be seen by our iso-elevation and iso-azimuth line calculations, this relates to 40°×40° of the visual field around the fovea (see results and Figure 4).

Superimposing iso-azimuth and iso-elevation lines illustrates that, although the stimulus directions were exactly perpendicular to each other, the iso-azimuth and iso-elevation lines formed an average angle of about 70° instead of 90°. This is in agreement with electrophysiological recordings conducted by Schmidt et al. [41] on the optic tectum of zebra finches, who revealed the representation of the horizon on the tectum to be tilted up by 20.6° against the real horizon. It is likely that this tilt has some functional implications since Plass [54] found that the lateral semicircular canal of the vestibular system also shows an upward tilt of about 20° in the zebra finch and thus exactly matches the visual field representation.

As mentioned above, the topographic representation which was revealed for the visual wulst by our experiments was much smaller than that obtained for the optic tectum. Since it was possible to expose the entire area of the visual wulst surface for the experiments, we are certain that we obtained the entire map in this case. While in the tectum 10° of visual space were represented on about 355 µm±118 µm in azimuth maps and 360 µm±100 µm in elevation maps, it was about 105 µm±50 µm (azimuth) and 190 µm±80 µm (elevation) in the visual wulst, which is just one sixth of the tectal space. Whether this size difference has functional consequences is not easy to determine. In general, an expanded representation in the cortex for a particular sensory area (i.e. a high magnification factor) means that a greater information density is concentrated in that sensory area, leading to finer discrimination thresholds [55]. The optic tectum is involved in stimulus location (e.g. [35]) which can probably be more precise if the neuronal representation occupies much space. As mentioned above, the role of the visual wulst is still under debate. Our finding of a topographical map emphasises the similarity with the mammalian visual cortex, which has long been allocated to stimulus identification. However, the discovery of two different informational streams starting from the primary visual areas casts doubts on an exclusive role of the geniculocortical pathway in identification processes (e.g. [56]).

It has been shown in a variety of birds and also for the zebra finch [57] that the visual wulst receives a projection from both eyes. At least in the zebra finch, however, the activation of the visual wulst by the ipsilateral component is very small. Our present experiments confirm earlier notions of a lack of substantial ipsilateral input to the visual wulst [26], [27]. Previous experiments in chicks [30], [58], [59] and pigeons [45] have suggested that such a lack of an ipsilateral representation might be a global feature of birds with laterally placed eyes. This indicates that the visual wulst of these birds is organised quite differently from that of owls, which have a big binocular overlap and a strong ipsilateral representation of visual space within the wulst [21]. Zebra finches and owls, however, may not express two types of wulst organisation. Instead, they may be representatives of the ends of a scale with varying influences of the ipsilateral eye on the visual wulst. Comparison of a number of avian species indicates that at least the size of the visual wulst correlates positively with the extent of the binocular visual field [60]. Thus, it is also possible that the ipsilateral influence on the visual wulst and even the occurrence of binocular neurons increases successively from laterally eyed birds like zebra finches to more frontally eyed birds like owls.

Our displacement experiments revealed that the frontal visual field is not represented in the visual wulst. This is in agreement with other studies. It was originally thought that the region of the retina receiving information from the frontal part of the visual field projects to the visual wulst, which receives information of both eyes and could thus combine the information from the binocular visual field. This had to be revised. As already mentioned above, the wulst in laterally eyed birds receives just a very small ipsilateral projection. Moreover, Remy and Güntürkün [61] identified the foveal region as the main source of information reaching the visual wulst. Güntürkün and Hahmann [62] confirmed this view by behavioural experiments in the pigeon indicating that the thalamofugal system is frontally blind to a large extent. Accordingly, zebra finches do not use binocular viewing for the identification of food. Instead, a grain is targeted by the fovea before the bird makes a head turn to direct the beak towards the food. This head turn is under visual control only in the first half of the movement while the second half is a precalculated ballistic movement [24]. Taken together, these findings suggest that the visual wulst representation may be used indeed for identification purposes.

Placement of the monitor at different azimuth angles suggested that the visual field is not evenly represented on the visual wulst. The frontal visual field does not seem to have any retinotopic representation of the visual wulst, and the region lateral to the fovea was mapped on less space compared with the region between 30° and the foveal direction (60°). An increased magnification of the retinotopic map between 30° and 60° azimuth may be explained by a higher density of retinal ganglion cells within the retinal region receiving input from this part of the visual field [50]. Accordingly, visual acuity in the pigeon is highest in the region between 30° and the foveal direction, is comparatively low lateral to the fovea, and declines sharply in the frontal visual field [63].

The vertical displacement experiment suggested that the region of the visual field activating visual wulst most effectively reaches from about 17° to 42° above the horizon in elevation, and that there is some interaction between different parts of the topographic representation. Such interaction could probably be due to the complicated receptive fields of at least some wulst neurons which have been shown in previous studies. Electrophysiological recordings in the pigeon [45] uncovered receptive fields with two distinct and highly excitable zones with additional inhibitory regions present in the field. According to Wilson [59], the discharge rate generated within each discharge centre differed individually in the chicken with various stimulus orientations.

Vertically elongated receptive fields with two excitatory regions, one covering the upper monitor part and the other the lower one, which either amplify or inhibit the activity of the upper excitatory region depending on the stimulus orientation, would explain our findings. A horizontal stimulus passing vertically over the entire monitor would then excite neurons receiving projections from both excitatory regions within their receptive fields. Stimulation of the lower excitatory region, however, would have an inhibitory effect on the responsiveness of the upper excitatory region. A vertical stimulus passing horizontally over the monitor on the other hand would elicit neuronal response from both excitatory regions. Stimulation of the lower excitatory region would have an amplifying effect on the responsiveness of the upper excitatory region. All these effects are in accordance with the results of our experiment with the split stimulation monitor.

The most exciting finding of the wulst experiments was the occurrence of multiple retinotopic representations. In about two-thirds of the zebra finches, more than one map was activated within the imaged wulst area. In some cases, the retinotopic colour coding indicated the weaker patch to be mirrored in relation to the stronger patch. We could not, however, find any anatomical basis for this finding, e.g. a lamina running through an activity patch, dividing it into two halves. In other cases (Figure 8), the second representation was entirely separated from the other one and rotated by about 90°. Although we as yet do not have identified the reasons why the representations are not identical in all cases, our results unequivocally show that there is, in contrast to previous accounts [19], [30], more than one visual representation within the visual wulst of birds.

Such compartmentation in birds also includes other sensory domains, as Manger et al. [64] have shown in the owl, where they revealed at least a double representation of the somatosensory surface. Multiple representational maps are thus not only found in mammals [65], they can also be demonstrated in birds. This again adds to the view that the wulst area of birds and the cortex of mammals are homologous structures [18].

Although we have, up to now, mostly emphasised the similarities between mammalian visual cortex and avian visual wulst, one has to be aware that there are also differences which require consideration. As topographical mapping is a general feature of visual systems and can even be found in insects [66] finding such visual field representations alone can never be a proof of homology. For owls, there are quite a lot of other neuronal features like ocular dominance and orientation selectivity which support the homology notion [19]. For birds with lateral eyes this has still to be worked out.

Another interesting difference is the size of the topographic map which is quite small in the zebra finch visual wulst compared to the mouse, although their brains are similar in size. In addition, there is also a difference in the range of magnification factor, which is about 40 to 50°/mm in the mouse [43] and 60 to 100°/mm for the zebra finch (this study). This is even more puzzling because the retina of birds comprises an approximately 10-fold higher density of photoreceptors and also an about 10-fold higher visual acuity compared to the mouse which should also result (see above) in a larger neuronal representation. Unfortunately, we have at present no interpretation for these differences between the zebra finch and the mouse brains. We do not, however, believe that this is a finding which severely endangers the idea of homology between the avian visual wulst and the mammalian visual cortex.

Comparison of the position of the map obtained by optical imaging with previous experiments in zebra finches [27] or the chicken [30], [58] indicates that the topographic maps appear to be at the caudal rim of the wulst area activated by stimulation of the contralateral eye with flashes. Although our results show some variation concerning the exact position of the retinotopic map which may be due to slight variations in the positioning of the stimulus monitor in the horizontal or to interindividual variability, there can be no doubt that the retinotopic maps which we obtained are not placed at the centre of the wulst area activated by flash stimulation. There may thus be other visually driven areas within the visual wulst which do not show retinotopy, but may, in accordance with findings in mammals, be with or without a more complex topographical mapping and/or of neurons not responding to moving bars [67]. The next step would therefore be to examine the visual wulst for such compartmentation into visual domains with different functional properties by using different kinds of visual stimuli such as stationary flashes, moving dots or more complicated visual scenes.

Last but not least, our optical imaging experiments may also contribute to the still unsolved question of whether the hyperpallial layers in birds are homologous to cortical layers in mammals [68]. In our experiments, recording depth was normally 500 µm. The recorded plane was parallel to the surface, but not really parallel to the laminae. However, we checked regularly whether focussing on 300 µm or 700 µm revealed differences. This was never the case. One could therefore presume that a tilted recording plane parallel to the lamina would also have revealed the same results. Together with the finding that the maps continued from HA over IHA to HD, this could be a strong hint towards a similarity of these wulst layers to cortical layers [69]. However, as can also be seen in Figure 5, the representation was very near to the border of the HA. If, in future experiments, one could demonstrate that a topographic representation at some place exists also at the lateral side of the lamina frontalis suprema, this would be an indicator in favour of the view presented by Medina and Reiner [68].

Taken together, our tectal results show by their consistent agreement with previous experiments the validity of the optical imaging method for recording in small birds. Our results concerning the visual wulst have also confirmed previous findings, have presented new ideas concerning the functional neuronal organisation of this area, and for the first time revealed more than one visual representation. Admittedly, many questions have remained open as, for example, the investigation of the total extent of the visual representation(s), the reason for the strong limitation of the representation to a stripe of the visual field above the horizon in elevation maps, or the unsolved question of lateralization of the wulst system which has been shown convincingly in a variety of studies. However, these unsolved questions may stimulate more work on this issue not only in our research groups.
